# "Atrial torsades de pointes" Induced by Low-Energy Shock From Implantable-Cardioverter Defibrillator

**DOI:** 10.1016/s0972-6292(16)30674-x

**Published:** 2013-09-01

**Authors:** Ilknur Can, Venkatakrishna Tholakanahalli

**Affiliations:** 1 Necmettin Erbakan University, Meram School of Medicine, Department of Cardiology, Konya, Turkey; 2 Veterans Affairs, Medical Center, Cardiovascular Division, Minneapolis, Minnesota, USA

**Keywords:** atrial torsades de pointes, implantable cardioverter defibrillator, low-energy shock

## Abstract

A 58 year-old-patient developed an episode of polymorphic atrial tachycardia which looked like "atrial torsades de pointes" after a 5J shock from implantable cardioverter defibrillator.

## Introduction

Electrical shock provided by implantable cardioverter-defibrilllators (ICDs) is an effective therapy proven to reduce mortality in patients with ischemic or non-ischemic cardiomyopathy [1,2]. Defibrillation shocks provided by the ICDs range from low-energy cardioversion for monomorphic ventricular tachycardias to high-energy shocks for ventricular fibrillation. Recently, there has been concern over the safety of low-energy cardioversions since it has been associated with high per-patient risk of ventricular fibrillation (VF) induction [3-5]. However, there has been no report on the atrial pro-arrhythmic effect of low-energy shock from ICDs.

## Case report

A 58-year-old male with a history of ischemic cardiomyopathy and decreased left ventricular ejection fraction (25%) received a DDD ICD in March 2004 for sustained ventricular tachycardia. The ICD was programmed for two VT zones and a VF zone. The slow VT zone was programmed for venticular rates over 146 beats/min and a fast VT zone (via VF) was programmed for rates 188-250 beats/min. In the fast VT zone, 2 burst antitachy pacing sequences were followed by ICD shocks (5J, 35JX4). The VF zone was programmed for rates over 188 beats/min.

**Figure 1:**
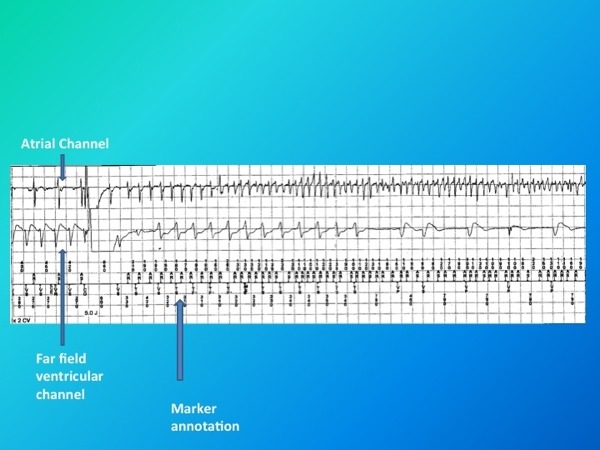
The upper tracing indicates near field atrial electrogram. The middle tracing indicates far field ventricular electrogram. The lower tracing indicates marker annotation channel. A 5 joule shock in the ventricle resulting in delay in subsequent 'P' wave resulting in polymorphic atrial tachycardia with constant shifting of p- wave axes.

**Figure 2:**
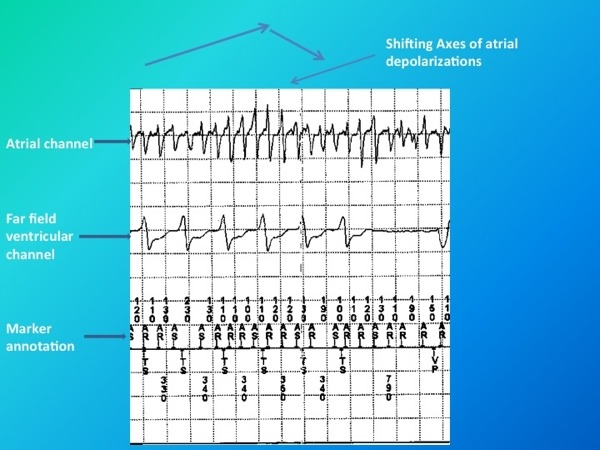
The upper tracing indicates near field atrial electrogram. The middle tracing indicates far field ventricular electrogram. The lower tracing indicates marker annotation channel. A close look up better reveals the constant shifting of axes of atrial depolarization (Atrial torsades de pointes).

**Figure 3:**
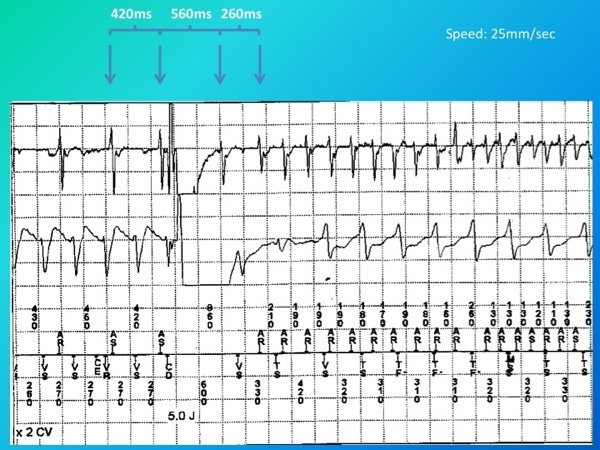
The upper tracing indicates near field atrial electrogram. The middle tracing indicates far field ventricular electrogram. The lower tracing indicates marker annotation channel. There is a short-long-short atrial activity resulted from 5J shock for ventricular tachycardia resulting in "atrial torsades de pointes"

In 2011, the patient experienced a syncopal episode followed by a single ICD shock. Upon ICD interrogation it was noted that patient had fast ventricular tachycardia with average ventricular cycle length of 320 ms (Figure 1). The unsuccessful burst sequences were followed by a 5-J shock which did not terminate the VT, but the cycle length of the VT got prolonged before spontaneous termination. Meanwhile, the atrial rhythm which was sinus before the 5J shock, degenerated into a polymorphic atrial tachyarrhythmia with shifting axis which looked like "atrial torsades de pointes" (tdp) (Figure 2). The rhythm was initiated with a short-long-short atrial sequence similar to ventricular tdp (Figure 3). The termination of this atrial rhythm was not captured by the device but the patient was in sinus rhythm when the device was interrogated 3 weeks after he received the 5J shock. The patient's previous shocks were re-analyzed which were all high energy shocks and did not reveal any atrial pro-arrhythmic effect. His medications included aspirin 325 mg daily, metoprolol succinate 100mg daily, valsartan 20mg twice daily, furosemide 40 mg once daily, atorvastatin 80 mg once daily and ezetimibe 10mg once daily. On physical examination his blood pressure was 126/76 mmHg, pulse 56 beats/min, respiratory rate 16/min, and temperature of 36.9 C. He didn't have any elevated jugular pulse, no crackles in lung bases, and had trace pedal edema. His laboratory examination showed serum potassium of 4.3 mmol/l, serum sodium of 137 mmol/l, serum chloride of 137 mol/l and creatinine of 1.3 mg/Dl.

## Discussion

Although previous clinical reports have demonstrated that low-energy shocks are associated with higher risk of VF induction [3-5], there has been no study on the atrial pro-arrhythmic effects of defibrillation shocks in ICD recipients. Our case has shown that a low-energy defibrillation shock induced atrial pro-arrhythmia, which was a polymorphic atrial tachycardia reminiscent of a "torsades de pointes" pattern. This is important since atrial arrhythmias are well known to lead to inappropriate ICD discharges leading to increased morbidity and mortality [6].

Defibrillation shocks have been associated with several adverse effects such as reinduction of VF, conduction disturbances, atrial and ventricular electrical and mechanical dysfunction (stunning), and induction of fatal arrhythmias from inappropriate shocks [7-11]. Particularly, the relationship between shock energy and postdefibrillation ventricular arhythmias in patients with ICDs has been investigated and found that postdefibrillation ventricular arrhythmias with a cycle length of ≤300 msec were more common after low energy shocks (<10J) [3]. In experimental models postdefibrillation ventricular arrhythmias occured due to shock induced spontaneous depolarizations of Purkinje or myocardial cells [7]. One of the explanations that shocks induce these adverse arrhythmic effects is through "shock induced electroporation"[12]. These are sarcolemmal holes disrupting cellular ionic homeostasis by causing loss of intracellular potassium and calcium overload. Calcium overload is known to induce automatic or triggered depolarizations [12]. Electroporation has also been shown to cause conduction block in animal models which sets the basis for reentrant arrhythmias [9,13].

Though ventricular proarrhytmia due to defibrillation shocks drew so much attention given its lethal consequences, there has been no report on the atrial pro-arrhythmic effect of ICD shocks. In an animal model, Fedorov et al.[14] investigated the effect of defibrillation shock on atria and found that atria are more vulnerable to electroporation and resulting conduction block and arrhythmia than ventricles. To the knowledge of the authors, our case is the first report of an atrial polymorphic tachyarrhythmia in a patient induced by a low-energy ICD shock. We have speculated that ICD shock might have induced this atrial arrhythmia by the so called "shock induced electroporation" in the atrium leading to calcium overload triggered activity and conduction block, all promoting "atrial tdp". Atria being more vulnerable to shocks might have been affected by this pro-arrhythmic effect of the ICD. But, the absence of similar atrial proarrhythmia with high energy shocks remains to be explained. Like the high energy shocks in the ventricle extinguishing all the fibrillatory waves, high energy shocks might induce a more uniform effect in the atrium preventing atrial tdp.

"Atrial torsades de pointes" has been first recognized in the congenital long QT syndrome (LQTS) which is characterized by a prolonged QT interval and a susceptibility to ventricular tachyarrhythmias known as torsades de pointes [15]. Apperantly, disordered repolarization that is characteristic of LQTS is not confined to ventricular myocardium but affects the atrial myocardium as well. In patients with LQTS, the duration of monophasic atrial action potentials were found to be prolonged and afterdepolarizations in the atrium, similar to ventricular tdp, preceded the polymorphic atrial tachyarrhythmias [16]. Seslar et al. [17], reported a case of LQTS patient whose ICD interrogation demonstrated an asymptomatic spontaneous episode of polymorphic atrial tachycardia with shifting axis reminiscent of a "torsades de pointes" pattern.

In conclusion, low energy defibrillation shocks can provoke not only ventricular pro-arrhythmic but atrial pro-arrhythmic events as well. In our case the episode was self limiting, but long lasting episodes have the potential to lead to inappropriate and even lethal ICD discharges. Thus, we'd rather not be tempted to program very low-energy cardioversions in ICDs given the various adverse effects.
